# Active experiences in volume management among peritoneal dialysis patients with long duration: A qualitative study

**DOI:** 10.12669/pjms.41.7.11472

**Published:** 2025-07

**Authors:** Xinmei Xing, Hong Zhang, Yao Huang, Xiangying Lv

**Affiliations:** 1 Xinmei Xing Department of Nephrology, Baoding No. 1 Central Hospital Baoding, Hebei, China; 2 Hong Zhang Department of Nephrology, Baoding No. 1 Central Hospital Baoding, Hebei, China; 3 Yao Huang Department of Nephrology, Baoding No. 1 Central Hospital Baoding, Hebei, China; 4 Xiangying Lv Department of Nephrology, Baoding No. 1 Central Hospital Baoding, Hebei, China

**Keywords:** Chronic kidney disease, End-stage renal disease, Illness perception, Peritoneal dialysis, Social support

## Abstract

**Objective:**

Peritoneal dialysis (PD) is one of the main renal replacement therapies for patients with end-stage renal disease, but many PD patients still experience significant volume overload (VO). Currently, there is a lack of research on VO in PD patients specifically focusing on the unique group of long duration PD patients. No studies have yet examined the impact of long dialysis vintage on VO from the perspective of research subjects. This study conducted in-depth interviews with long duration PD patients to explore their positive experiences in volume management.

**Methods:**

Using purposive sampling, 10 long duration PD patients from a nephrology department at a tertiary hospital in Baoding City, China, from September, 2024 to October 2024 were selected as interview subjects in this retrospective study. The phenomenological approach in qualitative research was employed, conducting semi-structured in-depth interviews with these patients using the “knowledge-attitude-practice” theoretical framework and incorporating a literature-designed interview guide.

**Results:**

Among the 10 interviewees, there were four males and six females aged 35 to 64 years. Data analysis extracted four main themes: illness perception, positive attitude, excellent compliance, and social support.

**Conclusion:**

The volume management in long duration PD patients was influenced by multiple factors. This study identified themes of illness perception, positive attitude, excellent adherence, and social support. The results may guide clinical healthcare professionals in helping PD patients actively address VO and implement targeted interventions to improve their quality of life.

## INTRODUCTION

Chronic kidney disease (CKD) is a progressive condition that can have a significant impact on the daily life and well-being of individuals.^1^ End-stage renal disease (ESRD) represents the final stage of CKD, where the kidneys are no longer able to function adequately to maintain health. Patients with ESRD require renal replacement therapy, such as hemodialysis, peritoneal dialysis (PD), or renal transplantation, to survive.^2^ PD is a convenient and flexible treatment option that can be performed at home, allowing patients to have more control over their treatment schedule and lifestyle.^3^ However, one common clinical issue that can affect patients undergoing PD is volume overload (VO), which occurs when there is an excessive accumulation of fluid in the body.^4^ Research has shown that up to 60% of PD patients may experience VO, which can lead to complications such as hypertension, left ventricular hypertrophy, and cardiovascular disease.^5^ Hence, managing fluid balance and preventing VO are critical aspects of care for PD patients to reduce the risk of cardiovascular complications and improve overall outcomes.

Despite the importance of fluid management in PD patients, there is a lack of comprehensive understanding of the factors influencing patients’ adherence to volume restriction. Long dialysis vintage refers to patients who have been on dialysis for an extended period, which may pose unique challenges and opportunities for managing fluid balance effectively. The Knowledge-Attitude-Practice model is a framework for changing human health behaviors. This model posits that knowledge is the foundation, attitude is the driving force, and behavior change is the ultimate objective. People acquire knowledge and skills through learning, gradually forming beliefs and attitudes related to them, ultimately leading to the generation of relevant behaviors.^6^

In light of these gaps in the literature, this study aims to explore the positive experiences of long dialysis vintage PD patients in managing VO. By conducting in-depth interviews with this specific patient population, the study seeks to uncover insights and perspectives that can inform interventions and strategies to improve volume management (VM) in PD patients. Understanding the positive aspects of VM from the patient’s perspective can help healthcare providers tailor their education and support to enhance patient engagement and adherence to fluid restriction (FS) guidelines. Ultimately, the findings from this study may contribute to the development of personalized and patient-centered interventions to optimize FS and cardiovascular health in PD patients.

## METHODS

The research subject involved a purposive sampling method, selecting PD patients from the Nephrology Department of the Baoding No.1 Central Hospital from September, 2024 to October 2024, with the sample size collected based on the principle of information saturation.

### Inclusion criteria:

- PD as the only dialysis treatment method.
- Dialysis vintage ≥ 10 years.
- Age ≥ 18 years.
- Stable condition, willing to participate in the study, and signed an informed consent form.

### Exclusion criteria:

- Unclear language expression or communication barriers.
- Mental disorders, cognitive impairment.
- Simultaneous hemodialysis.
- Combined with tumors, tuberculosis, severe trauma, and other diseases.

Data saturation was reached after interviewing 10 cases, and the basic information and lab tests of the interviewees were shown in Table-I and Table-II. The study was approved by the Institutional Ethics Committee of Baoding No.1 Central Hospital (Approval Number: 2024198, dated: August 30, 2024), and written informed consent was obtained from all participants.

**Table-I:** Patient general information data.

Patient	Sex	Age (Years)	Education Level	Employment	Marital Status	Primary Disease	Dialysis Duration (Years)	Living Alone	Self-care Ability	Medical Payment Methods
1	Male	64	College	No	Married	Chronic glomerulonephritis	11	No	Yes	Medical insurance
2	Female	39	High School	Yes	Married	Diabetic kidney disease	12	No	Yes	Cooperative medical care
3	Male	62	University	No	Married	Chronic glomerulonephritis	12	No	Yes	Medical insurance
4	Female	58	University	Yes	Married	Other	11	Yes	Yes	Cooperative medical care
5	Male	49	University	Yes	Married	Diabetic kidney disease	14	No	Yes	Cooperative medical care
6	Female	46	High School	Yes	Divorced	Chronic glomerulonephritis	11	No	No	Medical insurance
7	Female	35	College	No	Married	Diabetic kidney disease	11	No	Yes	Medical insurance
8	Male	52	College	No	Married	Diabetic kidney disease	12	Yes	No	Medical insurance
9	Female	47	High School	Yes	Divorced	Other	10	No	Yes	Cooperative medical care
10	Female	59	University	No	Married	Diabetic kidney disease	15	No	NO	Medical insurance

**Table-II:** Results of laboratory examination in hospital.

Variables	Hb (g/L)	Alb (g/L)	CREA (umol/L)	Sodium (mmol/L)	Potassium (mmol/L)	Calcium (mmol/L)	Phosphorus (mmol/L)	BNP (pg/mL)	LVEF (%)
Normal Range	110-150	40-55	41.0-73.0	137-145	3.50-5.30	2.11-2.52	0.85-1.51	0-100	45-55
P1	103.5	37.6	986.7	135.7	4.12	1.96	2.17	24589.8	48
P2	98.3	38.2	1126.3	136.9	4.56	2.45	1.68	12459.7	42
P3	118.4	34.9	1085.3	144.1	3.98	2.26	1.77	24896.5	41
P4	105.9	39.4	1286.7	135.7	4.17	2.17	1.69	14896.4	49
P5	112.3	38.1	863.7	138.4	3.76	2.36	2.14	12789.7	42
P6	125.7	42.6	958.4	141.4	4.17	1.99	1.78	29876.3	46
P7	108.6	39.4	1185.3	135.4	3.49	2.14	2.46	22756.4	39
P8	98.4	46.3	1275.3	132.6	3.67	2.31	1.78	19867.3	42
P9	108.3	33.7	1045.3	134.4	4.76	1.89	1.89	14789.3	41
P10	112.7	41.8	986.7	129.8	3.96	1.99	1.86	9878.5	38

***Note:*** Hb (hemoglobin), Alb (albumin), CREA (creatinine), BNP (brain natriuretic peptide), CRP (c reactive protein), LVEF (left ventricular ejection fraction

### Consent for publication:

Written informed consent was obtained from the patients for publication of this study.

Utilizing the phenomenological approach in qualitative research, in-depth semi-structured interviews were conducted with patients, using the “knowledge-attitude-practice” theoretical framework. Taking into account existing literature, the interview guide was designed with the following questions:

1. Are you aware of the content and importance of VO in the daily life of PD patients?
2. How do symptoms like edema and shortness of breath affect your daily life or work?
3. What do you do on a daily basis to maintain relative fluid balance?
4. How do you address any discomfort or symptoms that arise?
5. What do you believe are the causes of these symptoms?
6. In your opinion, what kind of help or guidance do you think is necessary?

The interviews took place in a quiet, private clinic room, using a one-on-one semi-structured in-depth interview style. Prior to the interview, the purpose, significance, and main content of the interview were explained to the research subjects. After obtaining consent, the interview process was recorded, and the researcher also took note of the subjects’ tone of voice, body language, and other non-verbal behaviors. Each interview lasted approximately 30 minutes. During the interview, the researcher created a relaxed atmosphere and employs questioning, listening, responding, probing, and repeating techniques to encourage the research subjects to express their true views and feelings. The interview ended when data saturation was reached and no new themes emerge.

Interview data analysis was conducted anonymously, with P1~P10 representing participants. The Colaizzi phenomenological data analysis method was utilized, involving repeated listening to recordings and transcription of the data into written text. Similar or related data were grouped together using a coding system to identify connections between them. Through analysis and inference, the main themes of the data were extracted. After the interview, the data was transcribed, coded, and analyzed by two individuals. In case of disagreements, group discussions will be initiated. It is important to establish a trusting relationship with the patient before the interview. For the initial interview, it is recommended to have an experienced interviewer present to assist and guide.

## RESULTS

**Fig.1:**
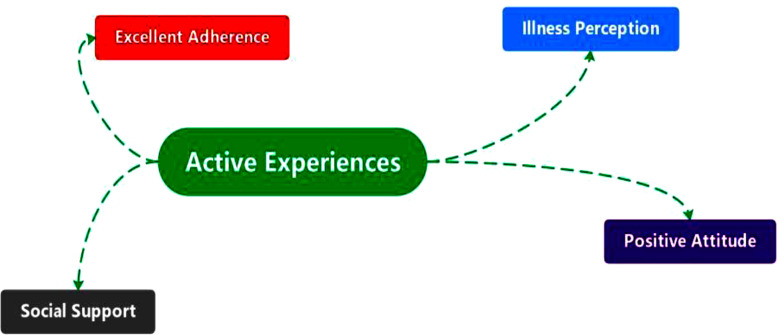
Four themes were identified for this study.

A total of 10 patients were retrospectively included in this study, with four males and six females, aged between 35-64 years, and with a dialysis vintage of 10-15 years (Table-I). Based on the theoretical framework of “knowledge, attitude, and practice,” this study conducted an analysis and synthesis to examine the positive factors related to VO in PD patients with long duration dialysis. Four main themes were identified through this process (Fig.1), which are detailed as follows.

### Theme-1: illness perception:

This interview revealed that three out of ten patients still had incomplete understanding of PD volume management, leading to inadequate handling of sudden issues and harboring fear of the disease. The majority of patients received good training from healthcare providers, which was greatly beneficial in managing their condition. *Patient-7:* “I have been on peritoneal dialysis for 11 years, so I am well aware of certain precautions for the disease. Swelling may be caused by consuming too much salt and drinking excessive water.” *Patient-2:* “A few days ago, I experienced severe swelling in my lower limbs, gaining five kilograms in weight. It was only during a follow-up visit that I realized it was caused by consuming too much salt and not controlling my water intake.” *Patient-3:* Is a working patient who eats out due to work commitments. As a result, their diet is not self-regulated, leading to excessive consumption of water and salt, which significantly worsens their swelling. However, by intensifying dialysis and controlling water intake, the swelling can be alleviated.

### Theme-2: positive attitude:

During the interview, eight patients were found to have a positive mindset and their condition was stable.

*Patient-1:* “I have accepted and gotten used to PD every day, feeling like a normal person with just an added routine. Dialysis is as routine for me as eating and sleeping.”

*Patient-5:* “I really enjoy chatting with cheerful friends on WeChat, it makes me feel light-hearted.”

*Patient-10:* “Although I initially disliked dialysis, I have now fully accepted it. I often take a one-hour walk every day and feel happy both physically and mentally, without any burden.”

### Theme-3: excellent adherence:

During this interview, seven patients showed excellent compliance. They were able to follow medical advice, communicate regularly with healthcare professionals, report problems promptly, and have good habits in managing daily diet and water intake. *Patient-2:* “If you don’t eat properly, your test results will not be good. The patient next to me during my last hospital stay ate and drank recklessly, leading to repeated hospitalizations. So, to avoid trouble, one must control their mouth firmly.” *Patient-4:* “During my last hospitalization, my heart rate was slow and serum potassium was high. The doctor prescribed medication, and controlling my diet improved my condition significantly.” *Patient-3:* “Look at that patient who refused to control his diet - he died after just two years of dialysis because he ate everything and didn’t listen to advice. Therefore, to live a quality life, one must follow medical advice and watch what they eat.” *Patient-10:* “Despite taking four types of blood pressure medication, my levels did not improve. Only when I changed to a light diet and reduced water intake did my blood pressure significantly improve.”

### Theme-4: social support:

The interview findings revealed that all 10 patients were able to receive some form of social support, but they were unable to access comprehensive support. Family caregiving support benefits PD patients in gaining positive experiences in capacity management. Medical support can enhance patients’ professional knowledge and technical skills. Government and community support can improve patients’ living environment and reduce treatment costs.

*P4:* “My son is very filial. Despite being busy with work on weekdays, he always come home on weekends to accompany me, chat with me, and take walks together. It makes me feel like life has purpose.” *P7:* “My spouse not only takes care of my daily needs, but also constantly encourages me to live well. He has never looked down on me as a worthless person.”

*P3:* “The Nephrology Department established a WeChat group for peritoneal dialysis communication, where patients regularly communicate with each other. When I encounter problems, medical staff promptly provide solutions, and there is also a public account that disseminates relevant knowledge. This has been extremely helpful for me.”

*P10:* “Hemodialysis for uremia was covered by major medical insurance, with strong support from the government. The out-of-pocket expenses on our end are minimal, making it affordable for our families.”

## DISCUSSION

This study utilized qualitative research methods to conduct in-depth interviews with PD patients with long dialysis vintage, in order to explore the positive factors that promote VO in MHD patients. Through a thorough analysis, it was found that enhancing patients’ illness perception, maintaining positive attitude, excellent adherence, and adequate social support can collectively improve the overall level of VM in PD patients with long dialysis vintage. This is the first study to explore patient-reported positive experiences in long-term PD volume management, particularly the synergistic role of social support and psychological resilience.

Numerous research studies have demonstrated a clear link between individuals’ illness perceptions and various psychological and clinical outcomes across different populations.^7^-^9^ This interview revealed that patients with PD, as their understanding of the disease deepens, experience significant relief from fear, leading to a positive shift in mindset and the acquisition of positive experiences in managing their condition. The increase in cognitive abilities also makes PD patients more receptive to reality, grateful for various forms of support, and able to reshape their mindset and experiences positively. Research showed that optimistic individuals were more actively involved in social life and better equipped to face challenges such as illness with a positive attitude.^10^ A positive mindset accelerates cardiovascular recovery after stressful events and reduces acute and chronic pain.^11^

The study revealed that 70% of PD patients had excellent adherence, with FS, while 30% had poor adherence. This non-compliance rate is consistent with the findings of Bossola M et al, indicating that PD patients’ adherence to fluid intake is suboptimal and needs improvement.^12^ In this study, 30% of PD patients did not prioritize FS enough, and 50% did not fully understand the reasons for FS, suggesting a lack of awareness and negative attitude among PD patients towards FS. This lack of understanding and negative attitude may be attributed to the limited human resources in PD centers, leading to inadequate follow-up management and education for these PD patients. Research demonstrated that knowledge, attitude, and practice were closely linked, and improving patient awareness could enhance their health attitudes and behaviors.^13^ Health education is an effective measure to enhance patient awareness and compliance.^14^ Limiting fluid intake is one of the most challenging treatment regimens for PD patients. Adherence is a multifaceted and complex issue influenced not only by objective factors but also by subjective factors such as patient psychology.^15^ The study found that all 10 PD patients received some degree of social support, mainly from their families.

However, the level of support from medical professionals and the community was limited, indicating a need for improvement in overall support. Family caregiving support benefits PD patients in managing their fluid levels effectively, while medical support can enhance patients’ professional knowledge and technical skills. Government and community support can improve patients’ living environment and reduce treatment costs. Comprehensive and systematic social support is crucial for the effectiveness of patient treatment, with roles from family, medical professionals, and the community being essential.^16^ During the treatment process, medical support is often the most direct. In addition to disease diagnosis and medication, nurses can strengthen health education and provide technical guidance, develop individualized care plans, and train PD patients and their families in caregiving skills, all of which contribute to a positive experience in managing fluid levels for PD patients.^17^

### Limitations:

Firstly, the research sample was gathered solely from one hospital, and the majority of participants were long duration PD patients. As a result, our sample may not accurately represent all PD patients, so it is recommended to include a more diverse sample in future studies. The single-center design and small sample size may limit generalizability. However, qualitative research prioritizes depth over breadth, and our sample size adhered to the principle of information saturation. Future multicenter studies are needed to validate these findings. Secondly, this research is a cross-sectional, observational study, meaning that the findings only capture a snapshot of the situation at that particular point in time. To understand the long-term effects of information on PD behaviors, further research should explore the longitudinal impacts. Thirdly, this study did not systematically document PD fluid types (e.g., dextrose or icodextrin concentrations) or diuretic prescription patterns. Given the retrospective design, detailed records of PD fluid types and diuretic prescriptions were unavailable. Future prospective studies should integrate prescription data to enhance analytical depth and incorporate such objective clinical data to comprehensively analyze factors influencing volume management. Finally, this study was qualitative in nature and suggests the need for additional quantitative or mixed methods research to provide further validation.

## CONCLUSION

This study explored the positive experiences of long duration PD patients in capacity management through in-depth qualitative interviews, highlighting four main themes: illness perception, positive attitude, excellent adherence, and social support. The findings of this study may serve as a valuable reference for healthcare professionals, as well as improve and enrich the capacity management models for PD patients. This helps individuals effectively cope with VO and implement targeted intervention measures to enhance their quality of life. Additionally, it was noted that there is a limited amount of intervention research on capacity management for PD patients in current clinical studies, indicating a potential direction for future work.
